# Unmasking Left Ventricular Diastolic Dysfunction: Pathophysiology, Diagnosis, and Treatment Strategies

**DOI:** 10.3390/medsci13030204

**Published:** 2025-09-22

**Authors:** Konstantina Vlasopoulou, Andreas Synetos, Nikolaos Ktenopoulos, Odysseas Katsaros, Leonidas Koliastasis, Anastasios Apostolos, Maria Drakopoulou, Konstantinos Toutouzas, Constantinos Tsioufis

**Affiliations:** 1 First Department of Cardiology, Hippokration General Hospital of Athens, National and Kapodistrian University of Athens, 11527 Athens, Greece; synetos@yahoo.com (A.S.); nikosktenop@gmail.com (N.K.); odykatsaros@gmail.com (O.K.); lkoliastasis@gmail.com (L.K.); anastasisapostolos@gmail.com (A.A.);; 2 School of Medicine, European University of Cyprus, 2404 Egkomi, Cyprus

**Keywords:** left ventricular diastolic dysfunction, diastolic dysfunction, echocardiography, tissue Doppler, strain imaging, heart failure, heart failure with preserved ejection fraction, HFpEF

## Abstract

Left ventricular diastolic dysfunction (LVDD) is characterized by impaired ventricular relaxation and increased chamber stiffness during diastole, resulting in increased left ventricular filling pressures. It represents a highly prevalent yet frequently underdiagnosed cardiac condition with significant clinical implications, serving as a major contributor to heart failure with preserved ejection fraction (HFpEF), particularly among elderly individuals and those with hypertension, diabetes mellitus, obesity, or coronary artery disease. Multiple studies have identified the progression of LVDD as a marker of adverse prognosis, associated with increased morbidity and mortality, highlighting the importance of early recognition and targeted therapeutic strategies to improve diastolic function and clinical outcomes. This review summarizes the pathophysiology, current diagnostic strategies, and treatment options for LVDD, emphasizing its importance in clinical practice.

## 1. Introduction

Left ventricular (LV) diastolic dysfunction (LVDD) results from impaired LV relaxation, reduced compliance (increased LV chamber stiffness), or a combination of both, leading to elevated filling pressures during diastole [1,2]. Population-based studies have shown a high prevalence of asymptomatic LVDD [3,4]. It is also recognized as a key mechanism in the pathophysiology of heart failure (HF) with preserved ejection fraction (EF) (HFpEF)—a condition that represents approximately 50% of all HF cases and is associated with significant morbidity, mortality, and healthcare burden [2,5,6,7,8]. Unlike HF with reduced EF (HFrEF), HFpEF is characterized by an increasing incidence. However, it is generally considered to have better survival, though with a negligible difference. Notably, mortality in HFpEF is more frequently attributed to non-cardiovascular (CV) causes, and the risk of non-CV death is greater in HFpEF than in HFrEF [5,6,9]. Nevertheless, compared to normal diastolic function, LVDD is associated with an increased risk of CV events and mortality [10]. LVDD and HFpEF are associated with older age, female sex, and a higher prevalence of hypertension, diabetes mellitus, obesity, atrial fibrillation (AF), and chronic kidney disease (CKD), while underlying coronary artery disease (CAD) is less common than in HFrEF [5,6,11,12,13,14,15,16,17,18,19].

Despite its clinical importance, LVDD often remains underdiagnosed due to its subtle and heterogeneous presentation. Patients may remain asymptomatic for extended periods or present with nonspecific symptoms such as exertional dyspnea and fatigue [5,6]. Furthermore, according to the recent American Society of Echocardiography (ASE) recommendations, accurate assessment of diastolic dysfunction is technically complex and requires a stepwise, multiparametric approach to minimize inconclusive cases [20].

Therapeutically, the management of LVDD and HFpEF remains challenging, as no drug has been shown to improve survival—unlike in HFrEF. According to the current guidelines, outcomes are only improved through aggressive management of underlying conditions and comorbidities, as well as treatment with sodium-glucose co-transporter 2 (SGLT2) inhibitors. These agents are the only drugs to have unequivocally reduced HF hospitalizations [6,21], although their precise role in modifying the underlying diastolic dysfunction is still under investigation.

The aim of this review is to elucidate the pathophysiological mechanisms underlying LVDD, summarize current diagnostic criteria and imaging strategies, and present the current therapeutic options based on the latest clinical evidence and guidelines.

## 2. Physiology of Diastole

**Figure 1:**
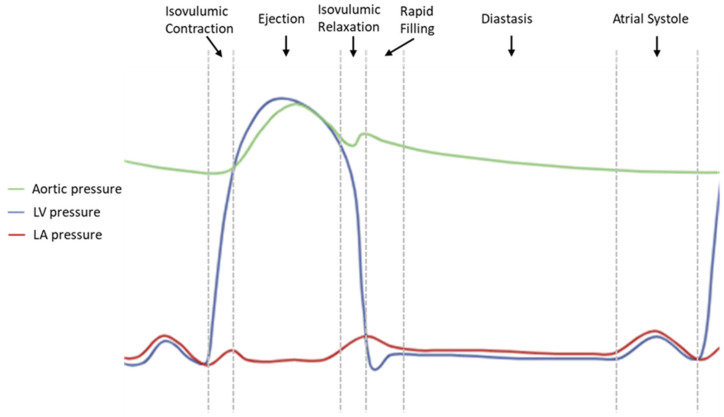
Left atrial (LA), left ventricular (LV), and aortic pressure curves depicting the phases of the cardiac cycle.

Diastole is the phase of the cardiac cycle extending from aortic valve closure to mitral valve closure. Clinically, normal diastolic function refers to the ability of the LV to relax and fill adequately with blood, thereby maintaining sufficient stroke volume at low filling pressures. Contrary to the traditional perception of diastole as a passive process, it involves both active, energy-dependent mechanisms and passive myocardial elastic recoil. Its efficiency is not determined by a single factor, but rather by a complex interplay of right ventricular (RV)–LV interaction, left atrial (LA) function, pericardial constraints, LV systolic properties, ventricular synchrony, coronary blood flow, and myocardial perfusion. Conventionally, diastole is divided into four phases: isovolumic relaxation, rapid filling, diastasis, and atrial systole (Figure 1) [22,23].

### 2.1. Isovolumic Relaxation

This phase begins immediately after aortic valve closure at the end of systolic ejection and ends with the opening of the mitral valve. It is defined by a rapid decline in left ventricular (LV) pressure (LVP), which, although decreasing, remains higher than LA pressure (LAP), keeping the mitral valve closed and resulting in no ventricular filling, and thus no change in LV volume. This phase is defined as the Isovolumetric Relaxation Time (IVRT) [22].

During systole, compression and torsion of the myocardium produce a progressive store of potential energy within the compressed titin of cardiomyocytes and extracellular collagen matrix, reaching its peak at end-systole. In early diastole, this energy is released through LV rapid elastic recoil and untwisting, aided by active myocyte relaxation, leading to a rapid increase in LV cavity dimensions and an equally steep decline in intracavity pressure. This nearly exponential LV pressure decay is characterized by the time constant Tau (τ), which is the most widely accepted index of LV relaxation. Normally, τ lasts 30 to 40 ms, and LV relaxation is nearly complete by approximately 3.5 τ. When relaxation is impaired, τ is prolonged and relaxation may not be completed until later in diastole; in extreme cases, incomplete relaxation persists until end-diastole, a state known as contracture [22,24,25,26].

### 2.2. Rapid Filling

As LV relaxation continues, the LVP falls below LAP, generating a suction effect that opens the MV, marking the onset of the rapid filling phase (RFP) and the end of the IVRT. Despite the initial increase in LV volume, LVP continues to decline due to ongoing myocardial relaxation. This generates an LA–LV pressure gradient, driving blood flow from the LA into the LV. This period constitutes the early part of the RFP, during which LVP reaches its minimum (LV_min_), corresponding to the descending limb of the LVP curve.

As LV volume increases toward its relaxed capacity, LVP gradually rises, diminishing the LA–LV pressure gradient. As LVP approaches LAP, this leads to deceleration of transmitral flow. This phase represents the latter part of the RFP, corresponding to the ascending limb of the LVP curve. Ventricular compliance is the main determinant of filling during this period; reduced compliance leads to a steeper rise in LVP, earlier MV closure, and a shortened RFP. Importantly, compliance influences filling throughout diastole, not only during the early phase [22,26].

During RFP, filling is facilitated by three physiologic processes: elastic recoil, active relaxation, and ventricular lengthening. Elastic recoil, as previously mentioned, facilitates LV relaxation and generates suction forces that contribute to the extent of early diastolic filling. Active relaxation is mediated by resequestration of calcium ions in the sarcoplasmic reticulum via the sarcoplasmic/endoplasmic reticulum calcium ATPase (SERCA) pump and its regulator protein phospholamban (PLB). Ventricular lengthening is facilitated by uncoupling actin–myosin cross-bridges. This low-force-generating state of the actin–myosin filaments increases the responsiveness of the LV muscle fibers to the lengthening load mediated by LAP, leading to LV enlargement and thus promoting LV filling [22,24,25,26,27].

Normally, during RFP, approximately 80 and 90% of total LV filling is accomplished, with the majority completed within the first 140 ms [22].

### 2.3. Diastasis

Diastasis represents the transitional phase between rapid filling and atrial systole. During this period, LVP gradually rises, while the LA–LV pressure gradient becomes minimal. As a result, transmitral flow declines significantly, and the MV leaflets adopt a semi-open or near-closed position. LV filling during diastasis is minimal and primarily sustained by passive flow from the pulmonary veins, modulated by LV compliance and the residual LA–LV pressure gradient. Diastasis constitutes the longest phase of diastole at normal heart rates but shortens progressively with increasing heart rate, usually disappearing at 90 to 100 beats/min [22].

### 2.4. Atrial Systole

LA contraction represents the final phase of diastole and terminates with MV closure. The increase in LAP during atrial systole actively propels blood into the LV, increasing filling volume. In young, healthy individuals, atrial contraction contributes approximately 20% of the end-diastolic volume, accompanied by a modest increase in LV end-diastolic pressure (LVEDP) of less than 5 mmHg. On the contrary, absence of LA contraction—as in AF—reduces LV stroke volume by approximately 20 to 30%. With advancing age, or in the presence of impaired relaxation and reduced compliance, residual LA volume after RFP increases. This residual volume is then injected into the LV via the Frank–Starling mechanism, contributing up to 40% of the total end-diastolic volume. LV filling during atrial systole depends on LV compliance, atrial contractility, and pericardial resistance [22,26].

## 3. Pathophysiology of Left Ventricular Diastolic Dysfunction

Impaired relaxation and increased ventricular stiffness—often coexisting—are the two central mechanisms of LVDD. These abnormalities can be attributed to intrinsic alterations within cardiomyocytes, extracellular matrix (ECM) remodeling, and maladaptive neurohumoral and endothelial signaling [27,28]. Collectively, these mechanisms contribute to elevated filling pressures, impaired ventricular filling, and ultimately HFpEF.

### 3.1. Impaired Relaxation

**Figure 2:**
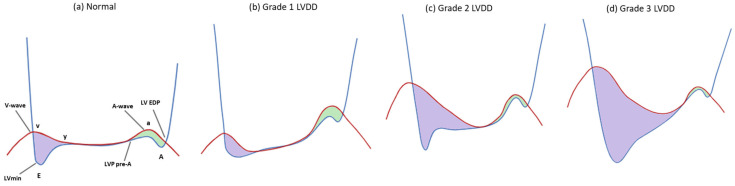
Simultaneous left ventricular (LV) and left atrial (LA) pressure recording showing early and late transmitral pressure gradients in each LV filling pattern. The blue line represents LV pressure, and the red line represents LA pressure. The purple shaded area indicates rapid filling phase, while the green shaded area indicates atrial systole. LVDD, left ventricular diastolic dysfunction; EDP, end-diastolic pressure; LVP, left ventricular pressure; LV_min_, minimum left ventricular pressure.

The initial stage of diastolic dysfunction is marked by impaired relaxation with preserved compliance, characterized by a prolonged τ and a longer IVRT. As relaxation slows, LVmin increases and the early LV–LA pressure gradient narrows, weakening suction-driven filling and decreasing early transmitral flow velocities. Nevertheless, since the ventricle remains compliant, overall LV filling pressure (LVFP) and, consequently, LAP stay within normal limits, and only a slight, clinically silent rise in LVEDP may occur—an early sign of diastolic dysfunction. This LV filling pattern corresponds to grade 1 LVDD (Figure 2) [22,29].

At the cellular level, any disruption in the processes of actin–myosin cross-bridge detachment and calcium ions resequestration in the sarcoplasmic reticulum can impair the rate of relaxation. Experimental studies consistently show prolonged Ca^2+^ transients, elevated resting Ca^2+^ concentration, and slower force decay in myocardium with diastolic dysfunction (e.g., hypertensive or HFpEF), confirming delayed myofilament inactivation [27,30,31].

Alterations in myofilament energetics also play a role. ATP is needed for cross-bridge detachment and active calcium resequestration. Reduced oxidative phosphorylation, impaired phosphocreatine-dependent ATP regeneration, or changes in the ADP/ATP ratio lead to slower cross-bridge cycling and prolonged diastolic tension [28,32].

Cytoskeletal remodeling contributes as well. Titin isoform switching from the more compliant N2BA isoform to the stiffer N2B isoform, as seen in hypertrophic and dilated cardiomyopathy, has been associated with impaired relaxation and increased myocardial stiffness [28,33,34].

Impaired myocardial relaxation typically presents in concentric LV hypertrophy due to hypertension or aortic stenosis, ischemic heart disease, and metabolic disorders such as diabetes and hypothyroidism. Regional mechanical asynchrony after myocardial infarction and increased afterload from arterial stiffness further delays relaxation [35].

### 3.2. Increased Ventricular Stiffness

While impaired relaxation affects early diastolic filling, increased stiffness mainly impairs during mid-to-late diastole, resulting in elevated diastolic pressures even at normal filling volumes (Grade 2 LVDD, Figure 2). As LV compliance deteriorates further, LV filling becomes restrictive, such that even low filling volumes produce markedly elevated diastolic LVP and, consequently, high LAP and LVEDP (Grade 3 LVDD, Figure 2).

**Figure 3:**
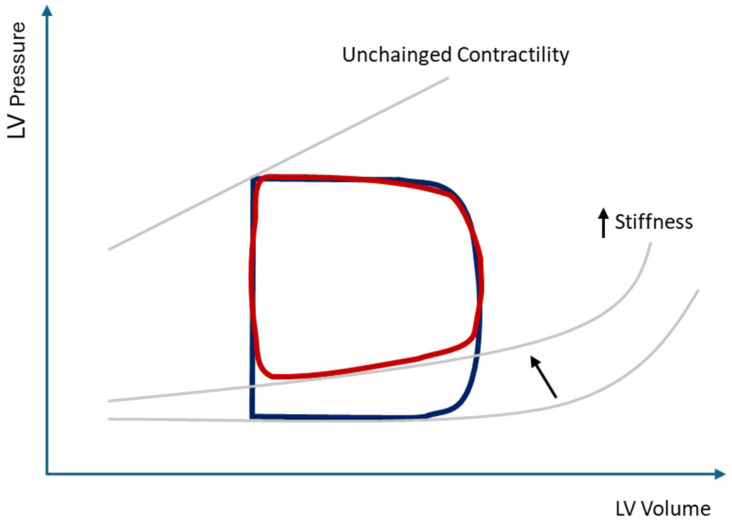
LV pressure–volume loop in LVDD (red line) compared with normal LV diastolic function (blue line). In LVDD, the end-diastolic pressure–volume relationship curve is displaced upward and leftward (arrow) due to increased chamber stiffness and elevated end-diastolic pressure, while LVEF remains normal. LV, left ventricular; LVDD, left ventricular diastolic dysfunction; LVEF, left ventricular ejection fraction.

While impaired relaxation with normal LAP prolongs IVRT, elevated LAP causes earlier MV opening and shortens IVRT. High LAP dominates early diastolic filling despite impaired relaxation, leading to increased early transmitral flow velocity followed by rapid LVP rise after LV_min_ and rapid deceleration of flow due to poor LV compliance. During diastasis, elevated LAP and atrial stiffness can sustain transmitral flow (L-wave). Increased diastolic LVP generates resistance to LA contraction, reducing forward flow and increasing backward flow into the pulmonary veins (PVs), transmitting pressure back to the pulmonary capillaries and contributing to pulmonary arterial hypertension (PAH) [22]. The net hemodynamic effect of these changes is an upward and leftward shift of the end-diastolic pressure–volume relationship, whereby filling occurs at the cost of increased LVFP (Figure 3) [2].

At the cellular level, titin isoform switching, along with post-translational modifications such as phosphorylation and S-nitrosylation, alters its biomechanical properties and increases myocardial stiffness [27,32,34,36]. In addition, pressure overload states are associated with microtubule accumulation, where increased density and disorganized architecture elevate cytoskeletal viscosity and mechanically impede sarcomere shortening and relaxation, thereby reducing ventricular distensibility [28,37].

Extracellular matrix remodeling, particularly excessive collagen deposition, plays a pivotal role. Collagen type I is highly tensile and resists stretch, while type III provides elasticity. In conditions such as chronic hypertension and diabetic cardiomyopathy, an excess of type I collagen, a reduced type I/III ratio, and increased cross-linking collectively impair ventricular compliance [27,36]. Myocardial fibrosis arises from an imbalance between collagen synthesis and degradation, driven by mechanical stress and neurohormonal activation. Fibrogenic pathways, particularly those mediated by the renin–angiotensin–aldosterone system (RAAS) and sympathetic nervous system, promote fibroblast activation and collagen deposition through transforming growth factor-beta (TGF-β) signaling. Conversely, collagen degradation is regulated by matrix metalloproteinases (MMPs) and their inhibitors (TIMPs). Several studies have suggested that dysregulation of this balance leads to excessive collagen accumulation, ventricular stiffening, and reduced myocardial compliance [27,28,38].

In addition to structural changes, endothelial mechanisms also modulate myocardial stiffness. Nitric oxide (NO), predominantly released in the subendocardial region and peaking during diastole, contributes to the dynamic, beat-to-beat regulation of myocardial relaxation and ventricular stiffness [28].

Infiltrative cardiomyopathies, such as amyloidosis, are also associated with LVDD. They are characterized by an inflammatory response to myocardial infiltration, which may progress to fibrosis, and they often involve increased ventricular wall thickness. Together, these changes contribute to increased myocardial stiffness, impaired diastolic function, and elevated LVFP [28,39].

## 4. Diagnosis of Left Ventricular Diastolic Dysfunction

### 4.1. Invasive Assessment of LVDD

Although cardiac catheterization remains the gold standard for the assessment of LVDD and LVFP, its routine use in clinical practice is limited due to its invasive nature. Invasive evaluation of diastolic function primarily involves calculating the time constant of LV relaxation (τ), which is considered prolonged when >48 ms, and the chamber stiffness constant (β), with abnormal values exceeding 0.015. LVFP is traditionally measured through direct assessment of LVEDP or indirectly via pulmonary capillary wedge pressure (PCWP), which reflects mean LAP in the absence of mitral stenosis (MS). An LVEDP > 16 mmHg or a PCWP > 15 mmHg is indicative of elevated LVFP [20].

### 4.2. Echocardiographic Assessment of LVDD

Echocardiographic evaluation of LV diastolic function is a key component of the routine evaluation of patients presenting with dyspnea or suspected. Given the high prevalence of LVDD in many CV diseases, clinical reports should include comments on diastolic function and/or LVFP whenever possible [20].

Since echocardiography cannot directly measure intracardiac pressures, it relies on indirect markers of abnormal diastolic pressures and LV pressure–volume relationships. While no single echocardiographic parameter has optimal sensitivity and specificity to directly assess diastolic function, a multiparametric approach—integrating indices of impaired LV relaxation, early-to-late diastolic filling ratios, and structural and functional alterations associated with elevated LA and LV diastolic pressures—can reliably evaluate diastolic function in most patients [20,40,41,42].

Although the 2016 guidelines from the ASE and the European Association of Cardiovascular Imaging (EACVI) aimed to simplify the assessment of LVDD and demonstrated good accuracy in estimating LVFP, a significant proportion of cases remain unclassifiable or indeterminate [1,43,44]. The recently updated ASE guidelines propose a more contemporary approach to evaluating LV diastolic function and diagnosing HFpEF, incorporating additional and emerging echocardiographic parameters to enhance diagnostic precision.

**Figure 4:**
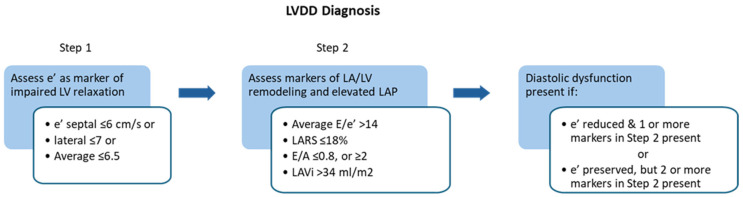
Algorithm for diagnosing LV diastolic dysfunction. Reprinted from Nagueh et al. [20], with permission from Elsevier. LVDD, left ventricular diastolic dysfunction; LA, left atrial; LV, left ventricular; LARS, left atrial reservoir strain; LAVi, left atrial volume index.

In contrast to the 2016 ASE/EACVI algorithm, the 2025 ASE recommendations for LVDD diagnosis adopt a two-step approach (Figure 4) [1,20]. The first step of the proposed algorithm relies solely on the assessment of mitral annular e′ velocity as a marker of impaired LV relaxation, given its established inverse relationship with τ [20,23,45,46]. This parameter demonstrates the highest feasibility and reproducibility in routine clinical practice. Its use, however, may be limited by the influence of regional wall-motion abnormalities in the sampled segment, significant mitral annular calcification (MAC), significant MR, prior MV repair or replacement, and pericardial disease [20,23,47]. Specifically, in constrictive pericarditis, the typical relationship between lateral and medial mitral annular e′ velocities is reversed, characterized by preserved septal e′ velocity and reduced lateral e′ velocity. This characteristic pattern is referred to as annulus reversus [48]. When discrepancies between septal and lateral e′ occur, an averaged value is recommended. Age-related thresholds further refine interpretation, with abnormal thresholds of average e′ velocity defined as <9 cm/s for individuals aged 20–39 years, <7 cm/s for those aged 40–65 years, and <6.5 cm/s for individuals over 65 years.

The second step incorporates indices of LAP and remodeling—E/e′ ratio, E/A ratio, LA reservoir strain (LARS), and LA volume index (LAVi) or LV mass index [20]. A reduced E/A ratio reflects impaired relaxation and diminished early diastolic filling [20]. Compared with the 2016 ASE/EACVI algorithm, two additional parameters—E/A ratio and LARS—are included, while peak TR velocity is excluded [1,20].

In the case of abnormal e′ velocity, at least one additional variable from the second step is needed to diagnose LVDD. If the e′ velocity is normal, then at least two abnormal indices from the second step are required. This updated algorithm reduces the number of indeterminate cases and improves diagnostic precision. Table 1 presents the abnormal cut-off values of the recommended variables [20].

It is crucial to recognize the inherent limitations of the main echocardiographic parameters, as these factors can influence their accuracy and applicability in various clinical scenarios.

The E/e′ ratio is commonly used to estimate LVFP and has been evaluated in several patient populations, including those with HFrEF and HFpEF. Intermediate and high values of the E/e′ ratio have been associated with increased risk of CV events [20,49,50,51]. Nevertheless, its use has several limitations. Since e′ velocity forms the denominator of the ratio, its accuracy decreases under conditions that impair e′ measurement [20,23,47,52]. Additionally, the E/e′ ratio poorly predicts mean LAP in patients with native left bundle branch block (LBBB) or RV/biventricular pacing, likely due to underestimation of septal e′ caused by suboptimal beam alignment from paradoxical septal motion [53]. Furthermore, a recognized “gray zone” exists between values of 8 and 14, where LVFP assessment is inconclusive [20]. In such cases, it is advisable to incorporate additional parameters, such as E/A ratio and IVRT, alongside the E/e′ ratio for a more accurate evaluation of LVFP [52].

**Figure 5:**
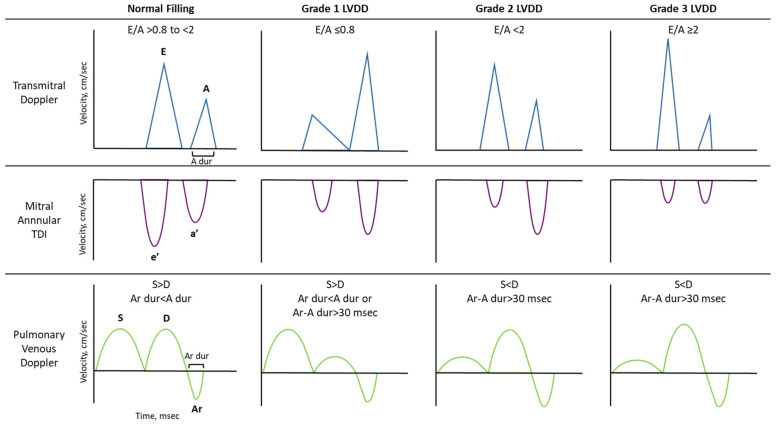
Doppler echocardiographic findings seen with different LV filling patterns. LVDD, left ventricular diastolic dysfunction; TDI, tissue Doppler imaging; A dur, A wave duration; Ar dur, atrial reversal wave duration.

Transmitral E/A ratio helps identify filling patterns (Figure 5). However, it can be misleading in the case of pseudonormalization (Grade 2 LVDD), and it is not useful in normal subjects. It is also highly preload- and heart-rate-dependent—not applicable in AF/atrial flutter—and decreases with age. In addition, E/A patterns can be difficult to interpret when E and A waves fuse and the E velocity at the onset of A is >20 cm/s, as the E/A ratio will be reduced. In ambiguous cases, it requires corroboration with other indices or maneuvers, such as Valsalva, since a decrease in the E/A ratio of ≥50% during this maneuver is highly specific for increased LV filling pressures [20,23].

LA strain analysis, used to evaluate LA phasic function, represents a newly introduced parameter in the main diagnostic algorithm of LVDD, with emphasis on LARS [20]. Several studies have shown that the LA strain has greater discriminative value than the well-established parameter LAVi in diagnosing LVDD [54,55], even in an early stage [56]. LARS shows a direct correlation with the degree of diastolic dysfunction and is inversely related to LVFP—lower LARS values indicate higher LVFP [57,58,59,60,61,62]. It is most accurate in detecting elevated LVFP in patients with reduced LV systolic function [57,58,59,60]. A LARS cutoff value ≤ 18% offers high specificity but may have low sensitivity in patients with normal left ventricular ejection fraction (LVEF) and preserved global longitudinal strain (GLS) for identifying elevated LAP [20,58]. Limitations include the requirement for specialized LA strain software, which is not universally available, and technical challenges in cases of poor image quality, mobile atrial septum, thin-walled LA, or inaccurate tracking of the mitral annulus. In addition, LARS is not recommended for patients with AF, significant MR, heart transplant recipients, or suspected LA stunning [20]. Finally, abnormal LARS cutoff values are age-dependent, decreasing with advancing age [20,60].

LAVi is a reliable parameter for assessing the chronic impact of elevated LVFP on the LA, but it correlates poorly with instantaneous LAP and has limitations in detecting early increases in LVFP [20]. In patients with preserved EF, recent findings show that the combination of LAVi with LARS significantly improves the detection of LV diastolic abnormalities and elevated LVFP compared with using LAVi alone [63]. Nevertheless, LAVi alone serves as an independent predictor of death, HF, AF, and ischemic stroke. Moreover, recent research has shown that, in patients with HFpEF, minimal LAVi may be a stronger predictor of CV outcome than maximal LAVi, highlighting its potential value in identifying patients at higher risk for CV events [64]. When using LAVi for LVDD evaluation, non-diastolic causes of LA enlargement—such as high-output states (e.g., anemia), heart transplants with biatrial technique, AF/atrial flutter, significant mitral valve disease, and physiological enlargement in well-trained athletes—should be excluded. Another limitation is that LAVi measurement can be technically challenging due to suboptimal image quality, foreshortening, or anatomical variations such as aortic aneurysms and large atrial septal aneurysms.

For the grading of LVDD and the estimation of LVFP, the first three echocardiographic indices that need to be assessed are e′ velocity, E/e′ ratio, and peak tricuspid regurgitation (TR) velocity, or preferably pulmonary artery systolic pressure (PASP) if right atrium (RA) pressure (RAP) can be measured. TR velocity ≥ 2.8 m/sec and PASP ≥ 35 mmHg in the absence of pulmonary parenchymal or vascular disease suggest elevated LAP.

The main limitations of PASP measurement are that it does not equal RVSP in the presence of pulmonary stenosis or right ventricular outflow tract obstruction (RVOTO) and cannot be accurately assessed in cases of severe TR with a low systolic RV–RA pressure gradient. Accurate PASP estimation also requires complete TR envelope acquisition, which may be improved by assessing the TR jet from multiple windows and using intravenous saline or ultrasound-enhancing agents to optimize the signal.

**Figure 6:**
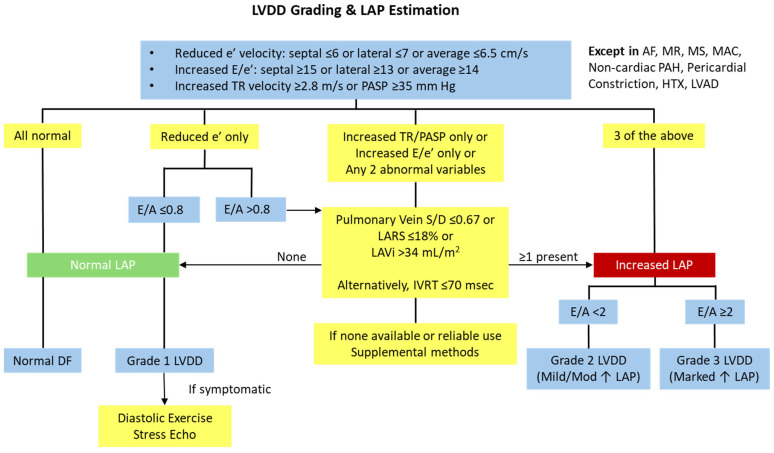
Algorithm for LVDD grading and LAP estimation for patients in sinus rhythm without severe primary MR, any degree of MS, moderate or severe MAC, MV repair, MV replacement, M-TEER, non-cardiac PAH, pericardial constriction, HTX, or LVAD. Reprinted from Nagueh et al. [20], with permission from Elsevier. LVDD, left ventricular diastolic dysfunction; LAP, left atrial pressure; TR, tricuspid regurgitation; PASP, pulmonary arterial systolic pressure; AF, atrial fibrillation; MR, mitral regurgitation; MS, mitral stenosis; MAC, mitral annulus calcification; PAH, pulmonary arterial hypertension; HTX, heart transplant recipients; LVAD, left ventricular assist device; S/D ratio, systolic-to-diastolic velocity ratio; LARS, left atrial reservoir strain; LAVi, left atrial volume index; IVRT, isovolumetric relaxation time; DF, diastolic function; MV, mitral valve; M-TEER, mitral-transcatheter edge-to-edge repair; ↑, increased.

If the first three variables in the algorithm—e′ velocity, E/e′ ratio, and PASP—are all within normal ranges, LAP is considered normal, and the diastolic function is classified as normal (Figure 6). On the other hand, the presence of all three findings—reduced e′ velocity, increased E/e′ ratio, and elevated PASP or peak TR velocity—is indicative of elevated LAP. Subsequently, if the E/A ratio is <2, diastolic dysfunction is classified as grade 2 (pseudonormalization), whereas if an E/A ratio ≥ 2 corresponds to grade 3 (restrictive). Grade 2 LVDD indicates mildly to moderately increased LAP and LVFP, while grade 3 LVDD reflects markedly elevated LAP and LVFP.

If the only abnormal finding among the initial three parameters is a reduced e′ velocity, and the E/A ratio is ≤0.8, this is consistent with grade 1 LVDD (impaired relaxation). However, if the E/A ratio is >0.8, further evaluation with additional variables is necessary, as elevated LAP may still be present in some of these patients. Similarly, if only the E/e′ ratio or PASP, or any two of the three parameters, are consistent with elevated LAP, additional variables should be evaluated. These include reduced LARS, increased LAVi, a decreased pulmonary vein systolic-to-diastolic (S/D) velocity ratio, or alternatively a shortened IVRT. In the case of none of these parameters being available or reliable, supplemental indices should be considered for evaluating LAP. Table 2 presents both the additional and supplemental parameters used for the estimation of LAP, along with their abnormal cut-off values. If none of the additional parameters are present, LAP is likely normal. However, if at least one of these parameters is present, it suggests elevated LAP, and diastolic function should be graded according to the E/A ratio, as previously described.

PV Doppler-derived parameters—such as the S/D ratio, atrial reversal (Ar) velocity and duration, and the Ar–A duration difference—can provide valuable hemodynamic information; however, obtaining adequate signal quality can be challenging. Notably, Ar velocity is absent in patients with AF [20]. Additionally, the correlation between PV-derived parameters and elevated LVFP is limited in patients with normal EF [20,65], AF, MV disease, and hypertrophic cardiomyopathy (HCM). Although the S/D ratio has good accuracy for identifying elevated mean LAP, in patients with normal EF, the S/D ratio can be >0.67 despite elevated LVFPs. In such cases, confirmation should be sought using IVRT or, if unavailable, other supplemental parameters.

Among the supplemental parameters, the presence of a triphasic transmitral inflow profile with mid-diastolic flow (L-wave) velocity ≥ 50 cm/s indicates markedly delayed LV relaxation and elevated LAP, reflecting a persistent LA–LV pressure gradient during diastasis. The L′ wave may also be visualized on the tissue Doppler imaging (TDI) trace between the e′ and a′ velocities [20]. In patients with this pattern, the presence of an L′ wave is associated with higher E/e′ ratios, elevated natriuretic peptide (NP) levels, and LA enlargement—findings consistent with advanced LVDD and increased LVFP [66]. This finding may rarely be seen in normal individuals with bradycardia, although, in such cases, the velocity is typically <40 cm/s [20].

Compared to the 2016 algorithm for LVDD grading and LVFP estimation, the 2025 recommendations introduce several key modifications to improve diagnostic accuracy. In the first step of evaluation, e′ velocity is used instead of E velocity, due to its stronger correlation with LV relaxation. LAVi, previously considered early in the assessment, is now evaluated in the second step, if needed. Additionally, the updated guidelines incorporate both additional and supplementary parameters to more effectively assess cases that would have been classified as indeterminate under the 2016 criteria [1,20].

Due to the limitations of the echocardiographic indices described above, the suggested algorithm can only be applied to patients in sinus rhythm who do not have AF, severe primary mitral regurgitation (MR), any degree of MS, moderate or severe mitral annulus calcification (MAC), MV repair, MV replacement, or mitral-transcatheter edge-to-edge repair (M-TEER). It should also not be applied to patients with non-cardiac PAH, pericardial constriction, heart transplant recipients (HTX), or LV assist device (LVAD). For these special populations, modified algorithms for the assessment of LAP have been proposed [20].

While current guidelines and available studies provide valuable insights, several limitations warrant consideration. Echocardiographic indices of diastolic function often show limited reproducibility in routine clinical practice, which may affect consistency in diagnosis and grading. Moreover, improvements in surrogate echocardiographic parameters, though encouraging, do not necessarily translate into meaningful clinical outcome benefits. Finally, uncertainty persists regarding the optimal approach to risk stratification in asymptomatic LVDD, highlighting the need for more robust prognostic tools and validated strategies to guide early intervention.

### 4.3. Left Ventricular Diastolic Strain Imaging

Longitudinal LV function is often impaired in patients with LVDD, even in the absence of overt systolic dysfunction. Both TDI and speckle-tracking strain imaging can detect this abnormality, with studies showing significantly reduced longitudinal and circumferential peak strain in HFpEF compared with hypertensive patients without HF and healthy controls [67]. Advanced strain-based indices—such as strain rate (SR) during isovolumic relaxation (IVR_SR_) and early diastole (e′_SR_)—derived from speckle-tracking GLS, have been proposed as alternatives to conventional e′ for estimating LV filling pressures [68]. Experimental studies also show significant associations between longitudinal and radial e′_SR_ and the extent of replacement fibrosis [69]. However, these methods are technically demanding, rely on high-quality imaging, lack standardization, and are less reliable in tachycardia due to frame-rate limitations [23].

Despite these challenges, strain-based indices—particularly the ratio of transmitral E velocity to IVR_SR_ (E/IVR_SR_) and the ratio of E velocity to e′_SR_ (E/e′_SR_)—have shown a direct relation with mean PCWP and independent prognostic value [68,70], with E/IVR_SR_ demonstrating a stronger association with PCWP than E/e′_SR_. E/IVR_SR_ also outperforms the traditional E/e′ ratio for detecting elevated LVFP in patients with segmental dysfunction or preserved EF [68]. Additionally, in a cohort of over 1000 post-myocardial infarction survivors, elevated E/e′_SR_ was associated with adverse outcomes, supporting the use of these indices as complementary tools for LVDD assessment and risk stratification [71].

LV GLS-derived parameters were not included in the recent ASE algorithm for LVDD and LVFP assessment. Nevertheless, they remain part of HFpEF evaluation criteria, as impaired LV GLS reflects subclinical LV dysfunction and is associated with worse outcomes in conditions like cardiomyopathies and left-sided valvular disease [20,41].

### 4.4. Diastolic Exercise Stress Echocardiography

Resting echocardiography frequently underestimates the severity of LVDD, as many patients with HFpEF develop elevated filling pressures only during exertion. Diastolic exercise stress echocardiography (ESE) is therefore useful in patients with grade 1 LVDD or normal resting LAP with unexplained HF symptoms or exertional dyspnea. It is also valuable for assessing symptomatic patients with indeterminate diastolic function or filling pressures at rest [20,72,73,74].

Diastolic ESE is typically performed using either supine bicycle or treadmill exercise echocardiography. Dobutamine stress echocardiography is not recommended [20,74], as dobutamine enhances left ventricular relaxation and early diastolic recoil, thereby increasing e′ velocity. This effect can lead to false-negative results by suggesting the presence of myocardial longitudinal diastolic functional reserve [74]. Unlike standard stress echocardiography for ischemia, the test typically requires only moderate exertion, although it can be combined with a conventional SE protocol with minimal additional time or resource demands.

During the exam, peak TR velocity should be measured first, immediately after wall motion assessment, followed by mitral inflow and mitral annular velocities once the E and A waves are separated. In most cases, when LVFP increases during exercise, it remains elevated for several minutes, allowing sufficient time to acquire Doppler velocities [20,74]. Similar information can be obtained using one minute of passive leg raising on a standard echocardiography table, although this approach lacks sensitivity [74,75]. Recently, detection of B-lines by lung ultrasound, indicating pulmonary congestion, has been proposed as part of diastolic stress echocardiography protocols, as it has been shown to improve risk stratification in these patients [76].

Normally, mitral e′ velocity typically increases by 3–5 cm/s with exercise. In patients with LVDD, however, this increase is minimal or absent. In these patients, E velocity increases while e′ remains unchanged, resulting in a higher E/e′ ratio [20]. Invasive studies have demonstrated a strong correlation between exercise E/e′ and invasively measured variables, indicating elevated LVFP under varying workloads, including supine bicycle exercise in the catheterization laboratory [57,72,77,78,79]. An average E/e′ ratio ≥ 14 (or septal E/e′ ≥ 15) combined with a peak TR velocity > 3.2 m/s indicates elevated LVFP [20,73]. HFpEF is considered likely when E/e′ is >14 and TR velocity is between 2.8 and 3.2 m/s, whereas HFpEF is unlikely if the E/e′ ratio is <14, regardless of peak TR velocity. An isolated increase in peak TR velocity does not confirm elevated LVFP [20].

Increases in the E/e′ ratio, as well as a PASP >50 mmHg or peak TR velocity >3.2 m/s during exercise, indicate a worse prognosis [78,80,81,82]. Data from a recent cohort of over 14,000 diastolic ESE studies showed that 17% of patients developed elevated LVFP with exercise, while 28% exhibited myocardial ischemia. Patients with elevated LVFP but no ischemia had worse outcomes than those with isolated ischemia [82]. Several studies highlight that diastolic ESE identifies a high-risk subgroup with strong specificity for diastolic dysfunction and adverse outcomes but limited sensitivity [72,78,80,81,82], reflecting patient selection and the absence of a universal gold standard for exercise hemodynamics. In a study of 498 patients with preserved ejection fraction, specifically referred for a diastolic ESE, 34% developed exercise-induced pulmonary hypertension, with only 29% of them reaching an E/e′ >15, confirming the test’s limited sensitivity. Prognosis was notably worse when pulmonary hypertension coexisted with E/e′ elevation, whereas isolated exercise-induced pulmonary hypertension without E/e′ increase did not carry the same adverse implications [81].

When exercise testing is not feasible, LARS may be a promising alternative, as it has been shown to identify patients likely to develop elevated LVFP during exercise [83]. A recent study tested a novel scoring system for diagnosing HFpEF based on ESE, utilizing three parameters—resting LARS, exercise septal E/e′ ratio, and B-lines—with promising results and possible prognostic value [84].

When ESE results are negative or inconclusive, but clinical suspicion for HFpEF still remains high, invasive hemodynamic assessment during exercise is recommended to confirm or exclude the diagnosis [20,85].

### 4.5. LVDD Assessment in Special Populations

AF is common in patients with diastolic dysfunction and HFpEF, but it presents several challenges for assessment due to a lack of organized atrial contraction, variable cycle lengths, tachycardia, and frequent LA enlargement. While various echocardiographic parameters have been proposed as markers of LVFP in AF patients with promising results [20], no single parameter is sufficiently reliable to be used alone. A multiparametric approach, on the other hand, yields only moderate accuracy in distinguishing normal from elevated LVFP [86].

For the evaluation of LVFP in AF, average values of multiple cardiac cycles should be used. For parameters like E/e′ and those relying on the timing of mitral E and e′ velocities, a dual Doppler probe may be used to capture both velocities within the same cardiac cycle and improve accuracy. Variability in mitral inflow with different cycle lengths can be indicative of LVFP, as patients with less beat-to-beat variability typically have elevated LVFP [20,87,88].

The first step of the modified algorithm for LAP estimation in patients with AF involves evaluation of E velocity, septal E/e′ ratio, peak TR velocity or PASP, and E-wave deceleration time (DT). LAP is classified as normal if none or only one of these parameters is abnormal, and as elevated if three or more are abnormal. In cases where two parameters are abnormal, further assessment of additional variables—LARS, pulmonary vein S/D ratio, and body mass index (BMI)—is warranted. If all additional variables are within normal limits, LAP is considered normal; if two or three are abnormal, LAP is classified as elevated; and if only one is abnormal, LAP is deemed indeterminate. It should be noted that several cutoff values for these parameters have been modified and are provided in Table 3 [20].

Another common finding, especially among the elderly, is MAC. It often coexists with hypertensive heart disease, aortic sclerosis, CAD, and CKD [20]. In moderate to severe MAC, the mitral orifice area is reduced, causing increased diastolic transmitral velocities and a decrease in lateral e′ velocity due to limited annular excursion. As a result, the E/e′ ratio may be elevated regardless of LVFP [47,52]. Moreover, LARS and diastolic LV SR do not reliably predict LVFP in this setting [89]. Instead, the E/A ratio and IVRT serve as more accurate predictors of LVFP in this population. Specifically, LVFP is generally normal when the mitral E/A ratio is <0.8, but elevated when it is >1.8. For intermediate values between 0.8 and 1.8, IVRT measurement is recommended. LVFP is typically normal if IVRT is ≥80 ms and elevated if <80 ms. A decision algorithm that combines these Doppler parameters has demonstrated accuracy in estimating LVFP in patients with MAC [52]. Furthermore, in a study of 206 consecutive patients, lateral and septal e′ velocities (LW-e′ and IS-e′), along with e′ velocities measured 2 cm distal to the annulus (LW-e′_2_ and IS-e′_2_), were assessed using TDI. It was found that adjusting LW-e′ by adding 1.6 cm/s and using IS-e′_2_ as a surrogate for IS-e′ may improve the accuracy of LV relaxation assessment in patients with MAC [47].

### 4.6. Assessment of LVDD by Cardiac Magnetic Resonance

Cardiac magnetic resonance (CMR) is considered the gold standard for quantifying cardiac chamber volumes, making it particularly well-suited for evaluating dynamic LV volume changes throughout the cardiac cycle, which can be transformed into filling curves using modern analysis software. In addition to volumetric assessment, CMR can directly measure blood flow velocities via velocity-encoded (phase-contrast) imaging, enabling evaluation of transmitral and pulmonary venous flow patterns similar to Doppler echocardiography. Myocardial tissue velocities can also be obtained, although current validation is limited to small studies. CMR offers a highly reproducible method for LA volume measurement, including minimal volume, though it consistently reports larger values than echocardiography [90]. Beyond volumes, CMR feature tracking allows derivation of LA functional indices, including strain, for the assessment of elevated LVFP [91]. The indices have demonstrated prognostic value for incident HF, in line with echocardiographic findings [92]. The MESA study further highlighted the prognostic potential of CMR-derived diastolic strain measures, showing that a novel relaxation index obtained by myocardial tagging independently predicted HF and AF over eight years, outperforming torsion and early diastolic SR and adding value beyond conventional risk factors, EF, and natriuretic peptides [93].

A distinctive strength of CMR lies in its ability to detect and quantify myocardial fibrosis—both focal replacement fibrosis, through late gadolinium enhancement (LGE), and diffuse interstitial fibrosis, via T1 mapping and extracellular volume (ECV) quantification [94,95]. Native T1 mapping captures intracellular and extracellular alterations without contrast, while post-contrast ECV imaging serves as a surrogate for interstitial expansion [96]. Studies have shown that ECV is slightly higher in HFpEF than in controls [95] but lower than in HFrEF [97]. These techniques can detect diffuse myocardial abnormalities not visible on LGE, providing incremental prognostic value in patients with or at risk of HF [95,98]. Nevertheless, their diagnostic utility for diastolic dysfunction remains experimental as studies report variable correlations between T1-based indices and invasive or echocardiographic measures of LVFP [98]. Biopsy studies show stronger associations than non-invasive parameters [99]. While these techniques hold promise for refining diastolic function assessment and risk stratification [95,100], further validation and standardization are required before routine clinical implementation [20].

## 5. Progression to HFpEF

LVDD may be present either with or without the clinical syndrome of HF and is associated with impaired prognosis [10,59,101,102]. It can occur in the setting of preserved systolic function and is present in almost all cases of systolic dysfunction [59]. The rate of LVDD progression to symptomatic HF remains variable and is mainly driven by the onset of hemodynamic alterations, the timing of which remains uncertain and may vary according to clinical context. A retrospective study of asymptomatic patients with moderate or severe LVDD on echocardiography found that only 12% developed overt HF within three years, and renal dysfunction was associated with this progression [103]. In contrast, among patients with diabetes, asymptomatic LVDD—particularly when accompanied by a septal E/e′ ratio > 15—was associated with more than a twofold increase in the incidence of overt HF over five years [104].

In the early stages of LVDD, elevations in LVFP and PCWP occur predominantly during exertion, leading to pulmonary congestion and reduced exercise tolerance. Over time, these pressures may become persistently elevated, giving rise to the full spectrum of HF symptoms that define clinical presentation [105].

Incorporating diastolic indices and GLS into echocardiographic screening protocols is a valuable strategy for identifying patients with LVDD who are at risk of developing incident HFpEF. These parameters capture a broader spectrum of cardiac dysfunction and detect early pathophysiological changes, enabling more accurate and timely prediction of HF risk [105,106]. In a study of 410 elderly asymptomatic patients with normal EF and at least one HF risk factor, echocardiographic assessment—particularly impaired GLS and increased LV mass—emerged as independent predictors of HF. Additionally, the presence of LA enlargement, LV hypertrophy, abnormal GLS, or elevated E/e′ was linked to more than a threefold increased risk of developing HF [107].

Interestingly, among echocardiographic indices used in HFpEF assessment, the LA stiffness index has demonstrated superior diagnostic performance compared with LARS alone, and similar or greater accuracy than commonly applied LVDD parameters in distinguishing HFpEF from non-cardiac causes of dyspnea [108,109]. The LA stiffness index—calculated as E/e′ divided by LARS—combines echocardiographic estimates of LAP with LA functional assessment to evaluate LA compliance in the context of LVDD. This index may reflect a more advanced stage of diastolic impairment and exhibits a closer correlation with adverse outcomes. Although a proposed threshold of >0.26 has been associated with increased risk of HF hospitalization or death in HFpEF patients with elevated LVEDP, further validation is required. Consequently, routine clinical use of LA compliance assessment is not currently recommended [109,110].

The progression of LVDD is not always unidirectional, as favorable reverse remodeling has been observed in some patients. In a large cohort of patients with dilated cardiomyopathy (DCM), reductions in LAVI over one year, including normalization in about one-third of cases, were associated with significantly improved outcomes, including a lower risk of death, heart transplantation, or HF hospitalization [111]. These findings emphasize the dynamic nature of diastolic dysfunction and the possible prognostic value of atrial remodeling in guiding management.

According to the most recent guidelines, the clinical diagnosis of HFpEF requires the presence of signs and symptoms of congestive HF, a LVEF of 50% or higher, and objective evidence of cardiac structural and/or functional abnormalities consistent with the presence of LVDD or elevated LVFP, including raised NP levels, in the absence of other cardiac or non-cardiac causes explaining the patient’s symptoms [5,6]. The initial step in the diagnosis of HFpEF involves recognizing the clinical HF syndrome. For patients with an indeterminate probability of HFpEF, diagnostic tools such as the HFA-PEFF diagnostic algorithm [41] and H2FPEF score [112] have been developed to aid in estimating the likelihood of HFpEF. In this setting, NPs may also guide the diagnosis [5,6,41].

## 6. Treatment Strategies

### 6.1. LVDD Treatment

Currently, no therapeutic strategies have been definitively shown to improve hard clinical outcomes in patients with asymptomatic LVDD. Management is therefore focused on prevention and risk factor control, aiming to slow progression to HFpEF by optimizing treatment of hypertension, diabetes, ischemic heart disease, obesity, and other cardiovascular comorbidities [6].

Only a limited number of large phase II interventional trials have assessed the impact of pharmacologic interventions on diastolic function using echocardiography as a primary or key secondary endpoint. For instance, the VALIDD trial examined whether blood pressure reduction with valsartan improved diastolic function. Improvement in e′ velocity was observed regardless of the specific antihypertensive used, along with better IVRT and systolic longitudinal tissue velocities. However, it remained unclear whether these changes reflected true myocardial diastolic improvement or were secondary to afterload reduction, which can accelerate relaxation [113]. The EXCEED trial compared intensive versus standard blood pressure control in patients with hypertension and LVDD, showing that the degree of blood pressure reduction was the primary driver of e′ improvement, with no significant difference between intensive and standard therapy. IVRT was the only diastolic indice with a clear incremental benefit in the intensive group, consistent with VALIDD [114]. The Aldo-DHF trial evaluated spironolactone in HFpEF, demonstrating improvements in E/e′ ratio, natriuretic peptide levels, LV mass index, and LV dimensions, but no gains in exercise capacity, symptoms, or quality of life. Although the effect on E/e′ persisted after adjusting for blood pressure changes, afterload effects could not be completely excluded [115]. These findings aligned with the TOPCAT trial, which ultimately showed no clinical outcome benefit of spironolactone in HFpEF [116].

Collectively, these studies highlight that echocardiographic improvements do not necessarily translate to symptomatic or prognostic benefit, emphasizing the need for integrated clinical endpoints when evaluating therapies targeting LVDD. Consequently, LVDD-specific therapies are not guideline-recommended, and treatment focuses on early detection, optimization of comorbidities, and prevention of progression to HFpEF [6].

### 6.2. HFpEF Treatment

#### 6.2.1. Pharmacological Therapy

At present, unlike in HFrEF, most clinical trials have faced challenges in identifying effective treatments for HFpEF. Large randomized controlled trials (RCTs) with angiotensin-converting enzyme inhibitors (ACE-Is) [117,118,119], angiotensin receptor blockers (ARBs) [118,119], mineralocorticoid receptor antagonists (MRAs) [116], and angiotensin receptor–neprilysin inhibitors (ARNIs) [120,121] have failed to meet their primary endpoints. While candesartan and spironolactone reduced HF hospitalizations, and sacubitril/valsartan showed a trend toward reduction, these observations are considered hypothesis-generating given the overall neutral results.

Several factors may explain these results. First, HFpEF represents a highly heterogeneous syndrome with diverse etiologies—ranging from hypertension and obesity to atrial fibrillation and microvascular dysfunction—making it difficult for a single pharmacologic intervention to exert uniform benefit. Second, many RCTs enrolled broad patient populations, often including individuals with borderline or improved LVEF, thereby diluting treatment effects. Third, the reliance on surrogate improvements in diastolic function or biomarkers has not consistently translated into outcome benefit, underscoring the challenge of targeting pathophysiological mechanisms that are not uniformly operative across patients. Finally, the natural history of HFpEF, with lower absolute event rates compared with HFrEF, limits statistical power to detect differences in mortality, meaning that modest benefits may go unrecognized. These limitations are also reflected in current clinical guidelines, which remain cautious in their recommendations for these therapies.

Accordingly, the American College of Cardiology (ACC)/American Heart Association (AHA)/Heart Failure Society of America (HFSA) 2022 guidelines state that ARBs, MRAs and ARNIs may be considered in selected HFpEF patients to decrease hospitalizations (Class IIb recommendation), whereas the European Society of Cardiology (ESC) 2023 updated guidelines do not provide comparable recommendations, reflecting the limited and largely surrogate-based evidence for these pharmacologic interventions [6,21].

Smaller studies with beta blockers have also failed to show improvement in prognosis or exercise capacity in HFpEF patients [122,123]. In the SENIORS trial, nebivolol significantly reduced the composite endpoint of all-cause mortality or CV hospitalization; however, only 15% of the participants had an LVEF over 50% [124].

In contrast, evidence-based interventions with demonstrated outcome benefits include SGLT2 inhibitors, as well as strategies aimed at symptom relief, including congestion and improvement of functional capacity.

Evidence from recent RCTs and subsequent meta-analysis studies demonstrates that SGLT2 inhibitors reduce the primary composite endpoint of CV death and HF hospitalization—mainly driven by a reduction in HF hospitalization—and improve symptoms regardless of diabetic status [125,126,127,128,129,130,131,132,133]. This has led to their inclusion in the treatment of patients with HFpEF, with a Class I recommendation in the ESC 2023 guidelines and a Class IIa recommendation in the AHA/ACC/HFSA 2022 guidelines [6,21]. Specifically, the EMPEROR-Preserved trial randomized 5988 patients with New York Heart Association (NYHA) class II–IV HF, LVEF > 40% and elevated plasma N-terminal pro-B-type NP (NT pro-BNP) concentrations to empagliflozin or placebo. Empagliflozin reduced the combined risk of cardiovascular death or hospitalization for HF in patients with HFpEF, regardless of the presence or absence of diabetes, although this effect was mainly driven by a reduction in HF hospitalizations, with no significant reduction in CV death [125]. The DELIVER trial enrolled 6263 patients with NYHA class II–IV HF who had an LVEF >40% at the time of recruitment or had an improved LVEF from ≤40% to >40%, along with elevated NPs levels. Dapagliflozin showed similar results to empagliflozin, reducing the combined risk of worsening HF or CV death, independently of diabetes status, once again mainly due to a reduction in worsening HF without a reduction in CV death [126].

Although these findings firmly establish SGLT2 inhibitors as the first pharmacologic therapy to improve clinical outcomes in HFpEF, uncertainty remains regarding their disease-modifying potential in LVDD. While no conclusive evidence exists that they alter the natural history of isolated LVDD, emerging data are encouraging. The DAPA-MODA study demonstrated that dapagliflozin, when added to optimized guideline-directed treatment in stable chronic HF patients, led to reductions in LAVi, LV mass, and NT-proBNP levels, suggesting a favorable impact on global reverse remodeling of cardiac structure [134]. Whether these surrogate improvements translate into long-term prognostic benefit in patients with LVDD, particularly in the absence of overt HF, remains to be clarified by future studies.

In addition, therapeutic management of HFpEF emphasizes relieving symptoms related to LVDD, including congestion. Thus, both the ESC and the ACC/AHA/HFSA guidelines give a Class I recommendation for volume management with diuretics [5,6,21]. Loop diuretics remain the agents of choice, although thiazide diuretics may be useful in patients with coexisting hypertension [5,6]. Due to the markedly steep diastolic pressure–volume relationship in advanced LVDD, even minor reductions in diastolic volume can result in substantial decreases in pressure and cardiac output. Therefore, careful titration is essential to avoid adverse effects such as hypotension and fatigue [28]. Importantly, while diuretics provide symptomatic relief and improve quality of life, they have not been shown to improve long-term prognosis or alter disease progression in HFpEF [5,6].

#### 6.2.2. Non-Pharmacological Therapy and Management of Comorbidities

Beyond pharmacologic therapy, lifestyle modification—including structured exercise and weight management—represents essential pillars in the treatment of LVDD and HFpEF [5,6,21]. Structured exercise training, especially individualized programs that combine aerobic and resistance modalities, has been shown to enhance endothelial function, increase peak oxygen consumption and exercise tolerance, improve diastolic function, and elevate overall quality of life, while also reducing hospitalizations related to HF [135,136,137,138,139,140]. Weight loss is a powerful modifier of disease course, especially in obese HFpEF patients, where caloric restriction or bariatric surgery has been associated with symptom reduction, enhanced exercise capacity, enhanced quality of life, and improved diastolic function [138,141,142].

Effective guideline-directed treatment of specific causes such as amyloidosis and contributing comorbidities is also critical to improve outcomes in HFpEF, as these conditions significantly influence disease progression [5,6,21].

Specifically, hypertension is the most prevalent cause of HFpEF, affecting 60% to 89% of patients. Individuals with HFpEF often exhibit an exaggerated hypertensive response to exercise and may present with acute pulmonary edema triggered by elevated blood pressure. Rigorous blood pressure control remains the cornerstone of managing left ventricular diastolic dysfunction. Guideline-directed medical therapy not only reduces the incidence of heart failure but also promotes regression of left ventricular hypertrophy, thereby improving overall cardiac structure and function [5,6,143,144].

Diabetes and insulin resistance contribute to myocardial injury and fibrosis in HFpEF. Antidiabetic therapies differ in their cardiovascular effects, and priority should be given to agents that are both safe and capable of reducing HF-related events. Among these, SGLT2 inhibitors have demonstrated improvements in clinical outcomes in patients with HFpEF and represent the preferred treatment for glycemic control [5,6,145].

CKD contributes to systemic congestion, inflammation, and neurohormonal activation, making optimal management—including blood pressure control, volume management, and renoprotective therapy—essential for improving outcomes in HFpEF. In patients with type 2 diabetes mellitus and/or CKD, in addition to traditional therapies with established cardiovascular benefit, SGLT2 inhibitors and MRAs, including finerenone, have been shown to reduce HF hospitalizations and improve clinical outcomes [21,146,147,148,149].

AF, highly prevalent in HFpEF, has a particularly detrimental effect on LA function and LV filling. The relationship between AF and HF is bidirectional, while AF can exacerbate HF, and HF itself increases the risk of AF development. Prognosis is less favorable when AF develops in patients with chronic HF, as this is associated with higher risks of stroke, hospitalization, and mortality. Management should include both rhythm- and rate-control strategies to optimize diastolic function, with rhythm control generally preferred when feasible. Catheter ablation is recommended as a therapeutic option for patients with paroxysmal or persistent AF whose HF symptoms persist despite optimal medical therapy, with evidence supporting its role in improving symptoms [5,6]. However, its efficacy and long-term outcomes remain insufficiently studied in individuals with HFpEF and AF, highlighting the need for further investigation in this population.

#### 6.2.3. Emerging and Investigational Strategies

As mentioned, myocardial fibrosis plays a key role in the pathophysiology of LVDD. Antifibrotic strategies focus on limiting extracellular matrix deposition or inhibiting profibrotic pathways, such as TGF-β signaling, that regulate collagen synthesis. Preclinical studies indicate potential improvements in cardiac function, although some approaches have been associated with toxicity. Targeting non-redundant pathways involved in myofibroblast activation appears particularly promising. However, large-scale clinical trials are still required to establish the safety and efficacy of these therapies in patients with HFpEF [150].

Emerging non-pharmacologic therapies are also under investigation. Device-based interventions, such as interatrial shunt devices, aim to decompress the left atrium and improve exercise hemodynamics in HFpEF patients. Early trials report favorable changes in cardiac structure and function in selected patients, though potential clinical benefit remains uncertain [151,152].

## 7. Future Perspectives

Early and accurate diagnosis of LVDD and HFpEF remains a critical unmet need, as late identification limits therapeutic efficacy. Emerging artificial intelligence (AI)-driven approaches show promise in automating echocardiographic measurements, integrating multiparametric data, and generating continuous diastolic function scores that capture disease across its spectrum. Validation of these models against invasive hemodynamics and outcomes is essential, while future integration into reporting systems could standardize and enhance routine clinical assessment [153,154,155,156,157].

The treatment of both LVDD and HFpEF remains challenging. In asymptomatic LVDD, no specific therapy has been shown to improve long-term outcomes, and current approaches emphasize careful control of cardiovascular risk factors to prevent progression. In HFpEF, the syndrome’s marked heterogeneity has likely contributed to the consistent failure of large randomized controlled trials to achieve their primary endpoints. Advancing toward more precise, pathophysiology-based phenotyping could enable the development of targeted therapies for distinct patient subgroups, thereby improving the chances of therapeutic success.

## 8. Take-Home Messages—Conclusions

LVDD results from complex interactions between myocardial, vascular, and systemic mechanisms that progressively impair relaxation and compliance, ultimately predisposing to HFpEF. While invasive hemodynamic assessment remains the gold standard, echocardiography—particularly when combined with strain imaging and exercise stress testing—serves as the cornerstone of non-invasive diagnosis. Cardiac magnetic resonance provides complementary insights into myocardial tissue properties and fibrosis, enhancing risk stratification. Despite these advances, challenges remain with reproducibility, indeterminate classifications, and the prognostic relevance of surrogate markers. Current management emphasizes strict control of comorbidities, alongside lifestyle modification and symptom relief with diuretics. SGLT2 inhibitors stand as the first class of drugs with consistent outcome benefits in HFpEF. Antifibrotic strategies and novel targeted approaches represent future directions but remain investigational. Collectively, progress in pathophysiologic understanding, refined diagnostic imaging, and evolving therapeutic strategies is gradually transforming the approach to LVDD and HFpEF, underscoring the importance of early detection and individualized care.

## Figures and Tables

**Table 1:** The recommended variables for identifying diastolic dysfunction and their abnormal cut-off values.

Parameters	Abnormal Cut-Off Values
e′ velocity	septal ≤ 6 cm/s or lateral ≤ 7 cm/s or average ≤ 6.5 cm/s *
E/e′ ratio	septal ≥ 15 or lateral ≥ 13 or average ≥ 14
E/A ratio	≤0.8 or ≥2 *
LARS	≤18%
LAVi	>34 mL/m^2^ ^#^
LV mass index	women >95 g/m^2^ men >115 g/m^2^ ^§^

* Age-specific cutoff values can also be considered to identify abnormally reduced e′ velocity or E/A ratio. ^#^ After excluding other causes of LA enlargement such as anemia, atrial flutter/fibrillation, significant MV disease, and athletic heart, ^§^ After exclusion of increased LV mass in athletes. LARS, Left Atrial Reservoir Strain; LAVi, Left Atrial Volume index; LV, left ventricular; MV, mitral valve.

**Table 2:** The recommended additional and supplemental variables for the estimation of LAP and their abnormal cut-off values.

**Additional Parameters**	**Abnormal Cut-Off Values**
LARS	≤18%
LAVi	>34 mL/m^2^
Pulmonary vein S/D ratio	≤0.67
IVRT	≤70 ms
**Supplementary parameters**	
PR end-diastolic velocity	≥2 m/s *
PAEDP	≥16 mmHg
Mitral inflow L-wave velocity	≥50 cm/s
Diastolic MR	Present ^#^
Ar velocity	>35 cm/s
Ar-A duration	>30 ms
E/A ratio with Valsalva maneuver	Decrease of ≥50%

* In the absence of pulmonary disease. ^#^ In the absence of advanced atrioventricular (AV) block, atrial flutter/fibrillation. LAP, Left Atrial Pressure; LARS, Left Atrial Reservoir Strain; LAVi, Left Atrial Volume index; S/D ratio, systolic-to-diastolic velocity ratio; IVRT, Isovolumetric Relaxation Time; PR, Pulmonary Regurgitation; PAEDP, Pulmonary Artery End-Diastolic Pressure; MR, mitral regurgitation; Ar, atrial reversal.

**Table 3:** The modified abnormal cut-off values of the recommended parameters used for the estimation of LAP in patients with atrial fibrillation.

Parameters	Abnormal Cut-Off Values
E velocity	≥100 cm/s
Septal E/e′ ratio	>11
LARS	<18%
Pulmonary vein S/D ratio	<1
BMI	>30 kg/m^2^

LARS, Left Atrial Reservoir Strain; S/D ratio, systolic-to-diastolic velocity ratio; BMI, body mass index.

## Data Availability

No new data were created or analyzed in this study.
